# Phage triggers spatial sporulation defense trade-offs

**DOI:** 10.1093/ismejo/wrag209

**Published:** 2026-08-13

**Authors:** Mengjie Wu, Zhonghua Cai, Jin Zhou

**Affiliations:** Institute for Ocean Engineering, Shenzhen International Graduate School, Tsinghua University, Shenzhen 518055, China; Institute for Ocean Engineering, Shenzhen International Graduate School, Tsinghua University, Shenzhen 518055, China; Institute for Ocean Engineering, Shenzhen International Graduate School, Tsinghua University, Shenzhen 518055, China

**Keywords:** phage infection, virospores, viral entrapment, phage-bacteria interaction, spore-forming bacteria

## Introduction

Bacteriophages (phages) are the most abundant and ubiquitous biological entities on Earth that drive bacterial mortality and shape evolutionary dynamics across every ecosystem [1]. In response, bacteria have evolved a multilayered arsenal of anti-phage defense systems, ranging from adaptive immune mechanisms such as CRISPR-Cas to innate barriers including restriction-modification systems and abortive infection [2]. Among these ancient and broadly effective strategies, endosporulation is remarkable. In this approach, the formation of metabolically dormant, stress-resistant endospores renders bacteria completely refractory to phage infection, even in genetically susceptible populations. For decades, sporulation has been primarily viewed as a last-resort survival strategy triggered by nutrient starvation [3–5]. Yet, in spatially structured environments where most bacteria reside, ranging from soil biofilms to host-associated microbial communities, phage infections spread as propagating fronts rather than through well-mixed liquid cultures. However, the spatial context underlying the reshaping of the evolutionary and ecological interplay between phage predation and bacterial sporulation has remained a critical gap in our understanding of phage-bacteria coevolution.

In a landmark study published in The ISME Journal, Măgălie *et al.* [6] uncovered a previously unrecognized collective defense mechanism: phage infection fronts directly trigger premature sporulation in adjacent bacterial populations, leading to the formation of a “sporulation ring” that halts phage spread, even in resource-replete environments. By using the spore-forming bacterium *Bacillus subtilis* as the model and the lytic phage SPO1, the study combined quantitative plaque assays, time-lapse microscopy, and spatially explicit mathematical modeling to dissect this dynamic interaction. Strikingly, the study revealed that phage plaques growing on lawns of wild-type, spore-forming *B. subtilis* were approximately 3-fold smaller than those growing on lawns of non-sporulating mutant strains. Critically, this reduction in plaque size was not driven by slower phage growth rates, rather by an abrupt, early termination of plaque expansion, an observation that defied the canonical model of sporulation as a starvation-induced response.

**Figure 1:**
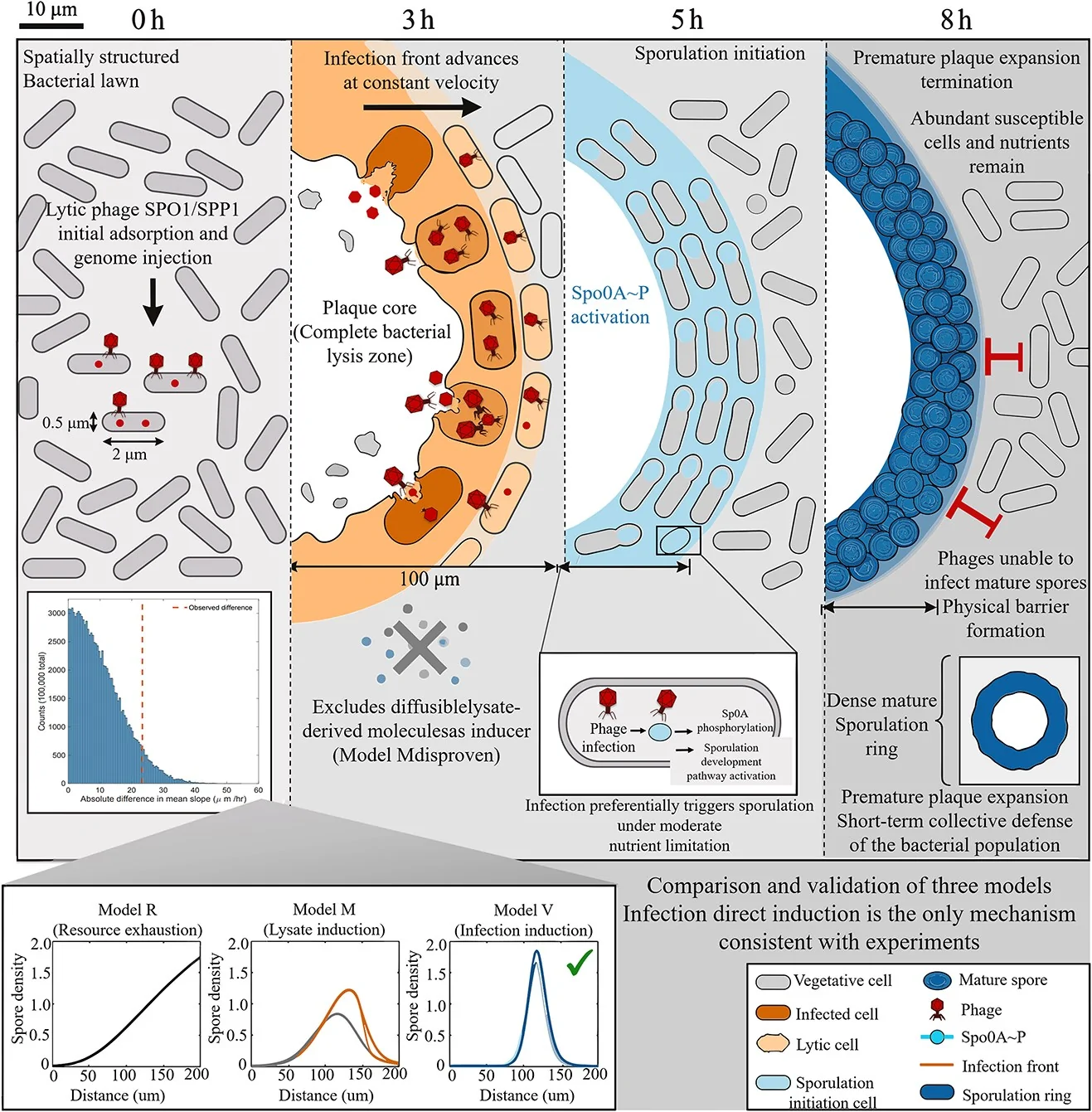
Mechanistic model of phage-induced premature sporulation in spatially structured *B. subtilis* populations. The resulting dense sporulation ring acts as a physical barrier that abruptly halts plaque expansion, serving as a short-term collective defense mechanism for the bacterial population. Only the infection-induced model (Model V) fully recapitulated the experimentally observed spore density distribution. Symbol definitions are provided in the figure legend.

Findings from high-resolution time-lapse imaging and mathematical modeling have revealed the spatiotemporal dynamics and mechanistic basis of phage-induced premature sporulation. Fig. 1 depicts the four consecutive stages of phage-bacterium interaction in spatially structured *B. subtilis* lawns. At 0 h (initial infection), the lytic phages SPO1 or SPP1 adsorb onto and inject their genomes into vegetative bacterial cells. At 3 h (infection front propagation), the phage infection front advances at a constant velocity, leading to the formation of a central plaque core in the completely lysed bacteria. At 5 h (sporulation initiation), direct phage infection triggers phosphorylation of the master sporulation regulator Spo0A (Spo0A ~ P) in cells at the infection front, initiating the sporulation developmental program long before global nutrient depletion occurs in uninfected regions. At 8 h (plaque termination), the formation of a dense, continuous ring of mature, phage-resistant spores occurs exclusively at the former infection front. This sporulation ring acts as an impenetrable physical barrier that abruptly halts further plaque expansion, even when abundant viable, susceptible bacteria and nutrients remain available outside the ring. Furthermore, to disentangle the molecular triggers of this phenomenon, three competing partial differential equation models corresponding to the three potential drivers were developed: resource depletion (Model R), diffusible lysate-derived signals (Model M), and direct phage infection (Model V). A quantitative comparison of the predicted spore density profiles demonstrated that only the infection-induced model (Model V) fully recapitulated the sharp, localized peak of sporulation at the plaque edge observed experimentally. Collectively, these data establish that direct phage infection, rather than indirect warning signals, triggers the formation of a sporulation ring that functions as a short-term collective defense for the bacterial population (Fig. 1).

This study fundamentally advances our understanding of phage-bacteria coevolution in multiple ways. First, it extends the established paradigm of starvation-triggered sporulation, demonstrating that phage predation acts as an additional potent and direct trigger of bacterial developmental differentiation, complementing prior reports of sporulation induced by predatory bacteria [7] and interspecies competition [8]. Second, it reveals a collective defense mechanism that is spatially coordinated, the sporulation ring acts as a population-level firewall that physically limits phage propagation, even at the cost of cells at the infection front. This distinguishes it from previously described community-level sporulation responses, which have primarily focused on the induction of sporulation by biotic cues [7, 8], rather than its spatial organization and protective function.

Looking forward, this study opens numerous exciting avenues for future research. The molecular identity of the diffusible signals that trigger premature sporulation remains unresolved, and its resolution will be critical to understanding this cross-kingdom interaction. Additionally, whether this mechanism is conserved across diverse spore-forming bacterial taxa and how it operates in complex, multispecies microbial communities require investigation. Finally, the exploration of ecological and evolutionary consequences of virospore formation in natural environments is only in infancy. Do these phage reservoirs shape the long-term stability of phage-bacterium coexistence? Can they facilitate horizontal gene transfer between bacterial populations or enable phages to switch hosts across environmental gradients?

In the decades-long quest to decode the evolutionary arms race between bacteria and phages, Măgălie *et al.* [6] uncovered a remarkable new layer of biological complexity. What, at first glance, appears to be a clear bacterial victory, halting phage invasion with a ring of dormant spores, reveals itself as a far more nuanced evolutionary trade-off in which bacterial defense and phage persistence are inextricably linked. This study not only fills a critical gap in microbial spatial ecology but also reminds us that, in the microbial world, the line between defense and capitulation is often far blurrier than it seems.

## Data Availability

Data sharing is not applicable to this article as no datasets were generated for the current study.
